# Androgen resistance in prostate cancer

**DOI:** 10.1038/sj.bjc.6604076

**Published:** 2008-01-08

**Authors:** J Waxman, S Ngan

**Affiliations:** 1 Department of Cancer Medicine, Division of Medicine, Faculty of Medicine, Imperial College London, Room 1014, Garry Weston Centre, Hammersmith Campus Du Cane Road, London W12 0NN, UK

The growth of prostate cancer is regulated by the androgen receptor (AR), which is a transcription factor that binds with steroid hormones, co-activators and co-receptors of AR, and translocates to the cell nucleus where the AR-complex initiates a number of androgen-related gene transcription events. In patients with prostate cancer treated with hormonal therapy, it is thought that with time and exposure to anti-androgens, AR mutates. This mutation causes a change in the regulation of prostate cancer cell growth, which may become independent of treatments aimed to decrease the availability of testosterone and dehydrotestosterone to the receptor.

There have been hundreds of AR mutations described, but one of the most commonly observed is at codon 877 (Sack *et al*, 2001). Mutation at codon 877 allows the tumour to become dependent for its growth upon the drugs used to treat it (Monge *et al*, 2006). In this situation, withdrawal of anti-androgens leads to a transient response in 20–40% of patients (Kelly and Scher, 1993).

As prostate cancer progresses, it is thought that there is further clonal evolution so that selection processes occur, leading to new ‘resistance’ to hormonal therapy, but this may not be an absolute resistance. It is likely that this selection process causes changes such that an individual's tumour may respond to further hormonal treatment; should that hormonal treatment be selective for the mutation that has developed. Thus it is likely that patients will respond to third- and fourth-line therapy with prednisone or oestrogens or dexamethasone or hydrocortisone or other steroidal agents. It is superficially and relatively straightforward to understand why these responses should occur. This is because the cellular receptors for these steroids are members of a supergene family sharing considerable structural homology (Boonyaratanakornkit and Edwards, 2007).

In this context, it is of interest to review the publication by Shamash *et al* reported in this current edition of the *British Journal of Cancer*. In their report, Shamash *et al* describe 56 patients with prostate cancer who were treated with sequential hormonal therapy and then, on progression, with a novel chemotherapy regimen. During the period of treatment with chemotherapy, hormonal therapy was discontinued. At the end of the treatment, 43 patients were re-challenged with hormonal therapy. Twenty-two of these patients were non-castrate at the end of treatment and 21 castrate. Sixteen of this group of 43 patients (37%) then went on to have a further PSA in response to either an LHRH agonist given with an anti-androgen or to a combination of diethylstilboestrol and dexamethasone. The responses were transient, but what is remarkable is that 12 out of 28 patients, who were treated with hormonal therapy following chemotherapy, went on to have another transient PSA response after progression on maximal androgen blockade (Shamash *et al*, 2007).

It is not clear whether these responses seen were due to induction of hormonal sensitivity through an unknown mechanism or due to regrowth of hormone-sensitive clones of cells which were previously suppressed by the original anti-androgen therapies. Regardless of mechanism, this paper has given oncologists and their patients reasons to be grateful for a further option in the treatment armamentarium, and this has been confirmed by Cox and Sundar in two patients in correspondence published in this current edition of the *British Journal of Cancer* (Cox and Sundar, 2007).
